# Linguistic style as a digital marker for depression severity: An ambulatory assessment pilot study in patients with depressive disorder undergoing sleep deprivation therapy

**DOI:** 10.1111/acps.13726

**Published:** 2024-07-10

**Authors:** Lisa‐Marie Hartnagel, Ulrich W. Ebner‐Priemer, Jerome C. Foo, Fabian Streit, Stephanie H. Witt, Josef Frank, Matthias F. Limberger, Andrea B. Horn, Maria Gilles, Marcella Rietschel, Lea Sirignano

**Affiliations:** ^1^ Mental mHealth Lab, Institute of Sports and Sports Science Karlsruhe Institute of Technology Karlsruhe Germany; ^2^ Department of Psychiatry and Psychotherapy, Central Institute of Mental Health, Medical Faculty Mannheim University of Heidelberg Mannheim Germany; ^3^ Department of Genetic Epidemiology in Psychiatry, Central Institute of Mental Health, Medical Faculty Mannheim University of Heidelberg Mannheim Germany; ^4^ Institute for Psychopharmacology, Central Institute of Mental Health, Medical Faculty Mannheim University of Heidelberg Mannheim Germany; ^5^ Neuroscience and Mental Health Institute University of Alberta Edmonton Alberta Canada; ^6^ Department of Psychiatry, College of Health Sciences University of Alberta Edmonton Alberta Canada; ^7^ Hector Institute for Artificial Intelligence in Psychiatry, Central Institute of Mental Health, Medical Faculty Mannheim University of Heidelberg Mannheim Germany; ^8^ University Research Priority Program (URPP) Dynamics of Healthy Aging, Healthy Longevity Center University of Zürich Zürich Switzerland

**Keywords:** ambulatory assessment, depression, ecological momentary assessment, experience sampling, LIWC

## Abstract

**Background:**

Digital phenotyping and monitoring tools are the most promising approaches to automatically detect upcoming depressive episodes. Especially, linguistic style has been seen as a potential behavioral marker of depression, as cross‐sectional studies showed, for example, less frequent use of positive emotion words, intensified use of negative emotion words, and more self‐references in patients with depression compared to healthy controls. However, longitudinal studies are sparse and therefore it remains unclear whether within‐person fluctuations in depression severity are associated with individuals' linguistic style.

**Methods:**

To capture affective states and concomitant speech samples longitudinally, we used an ambulatory assessment approach sampling multiple times a day via smartphones in patients diagnosed with depressive disorder undergoing sleep deprivation therapy. This intervention promises a rapid change of affective symptoms within a short period of time, assuring sufficient variability in depressive symptoms. We extracted word categories from the transcribed speech samples using the Linguistic Inquiry and Word Count.

**Results:**

Our analyses revealed that more pleasant affective momentary states (lower reported depression severity, lower negative affective state, higher positive affective state, (positive) valence, energetic arousal and calmness) are mirrored in the use of less negative emotion words and more positive emotion words.

**Conclusion:**

We conclude that a patient's linguistic style, especially the use of positive and negative emotion words, is associated with self‐reported affective states and thus is a promising feature for speech‐based automated monitoring and prediction of upcoming episodes, ultimately leading to better patient care.

**Significant outcomes**

- The use of positive and negative emotion words is associated with momentary depression severity assessed in major depressive disorder patients undergoing sleep deprivation therapy.
- Our findings generalize to other affective states assessed (positive and negative affective states, calmness, valence, energetic arousal).
- The frequency of positive and negative emotion words as a behavioral marker of depression severity is promising for a future speech‐based depression monitoring system.

**Limitations**

- Our sample size is limited and future studies are necessary to evaluate replicability.
- The task instructions might have biased word use. The development of a standardized instruction in the field would be helpful.

## 1 INTRODUCTION

Major depressive disorder (MDD) is a major health challenge and often manifests itself in a recurring or chronic condition.^1^ In the absence of biomarkers,^2^ guiding diagnosis and treatment has traditionally relied on subjective self‐report measures such as questionnaires and interviews by mental health professionals.^3^ As these are administered at sporadic points in time, they are (a) prone to retrospective bias,^4^ and (b) even full episodes might be missed.^5^ Patterns such as moment‐to‐moment fluctuations in symptoms which might be central regarding potential triggers and prodromal warning signs will remain undetected.^5^

With the features of near real‐time, continuous, active and passive data collection, the use of ambulatory assessment (AA) is advantageous as it reduces retrospective recall bias and increases ecological validity.^5^, ^6^ AA involves the assessment of momentary self‐reported symptoms and behaviors assisted by personal digital devices while patients perform their normal daily activities in their natural environment.^7^ One idea to reduce reliance on self‐reports is to derive objective parameters from speech. Two different streams of parameters have been used in the past: acoustic and linguistic features. Multiple studies revealed differences in the acoustic dimension of speech (e.g. pitch, jitter) between depressive and non‐depressive states^8^ or between depressed and healthy individuals (for reviews see^9^ and^10^). Leveraging the development of natural language tools such as the Linguistic Inquiry and Word Count (LIWC^11^), linguistic style or the choice of words in association with MDD is also investigated. Pennebaker and colleagues^12^ state that individuals' everyday word use, interpreted as a behavioral manifestation of thoughts and emotions, can reveal affective and cognitive processes characteristic of mood disorders.

As the potential linguistic feature space is extensive, researchers either pursue a brute‐force approach (e.g. References [13, 14]) or inform their feature selection by theoretical considerations of MDD (e.g.^15^, ^16^). With regard to the latter, in many studies the use of (1) positive and negative emotion words, (2) first person singular pronouns, and (3) past tense words were analyzed. Traditional theories of depression such as Beck's Cognitive Model of Depression^17^ or heightened self‐focus theories^18^ suggest such word use. However, empirical results are often mixed which might be due to the variety of methodological approaches and samples. Improved depressive states have been found to be associated with: (i) heightened use of positive emotion words and little use of negative emotion words (e.g.^14^, ^15^, ^16^), but also greater use of both positive and negative emotion words^19^; (ii) little use of first‐person singular pronouns (review by References [14, 20, 21]), but also opposite effects^22^ and null‐effects^16^; (iii) little use of past tense (e.g. References [23, 24]), but also the contrary.^19^

In the light of future automatic everyday monitoring systems, which shall monitor the change of trajectories of patient diseases and prevent upcoming episodes, two approaches are of particular relevance: (1) longitudinal studies exploring within‐person effects and (2) data collection in the field instead of controlled therapy sessions. According to current findings, only two studies meet these criteria.^13^, ^22^ In the first study, 120 linguistic and acoustic features extracted from mental health patient recordings were analyzed.^13^ Positive and negative emotion words and overall speech features were associated with global clinical assessments and depression subscores. However, only 15% of the sample were MDD patients and machine learning results refer to the full set of speech features. In another study, speech samples from MDD patients were aggregated to pre‐ and post‐treatment assessments.^22^ Decreases in negative emotion words, no difference in positive emotion words and increases in first person pronouns for post‐ versus pre‐treatment assessment were identified.

To inform the development of an automatic monitoring tool, robust studies in the natural environment of patients during and between state of the art assessed depressive episodes are needed. Those that exist exploit the potential of AA only weakly, for instance by aggregating multiple assessment points. Additionally, while there is a growing body of work on *inter*individual differences, studies on longitudinal *intra*individual differences during states of different depression severities are lacking. Understanding the fluctuations that occur may help to get a clearer clinical picture and provide warning of impending episodes.

With the present study, we aim to overcome the above‐mentioned limitations. Based on the available evidence, the linguistic style of speech of clinically diagnosed MDD patients in association with their reported depressive state has not yet been compared longitudinally during treatment with high temporal resolution. We analyzed a dataset of speech samples and concomitant self‐reported momentary affective states collected longitudinally via AA from MDD patients undergoing sleep deprivation therapy (SDT) in an exploratory way. SDT is a chronotherapeutic intervention that can rapidly improve depression severity.^25^ For our linguistic study, this is advantageous, as maximum variance in depressive symptomatology is gained within a short period of time, enabling to observe associated modifications in linguistic style within days. Finally, instead of averaging data from multiple sampling points into composite measures, we stay on the most granular level of our data collection (multiple data points per day per person) expecting to capture the dynamic ebb and flow of affective states.

To limit alpha error inflation and informed by the existing literature, we decided to include four LIWC word categories that closely resemble psychopathological phenomena. Accordingly, we formulated four hypotheses regarding associations between LIWC categories and concomitantly reported affective momentary states: With lower reported depression severity, we expected (1) more words in the LIWC category *positive emotion words*, (2) less words in the LIWC category *negative emotion words*, (3) less words in the LIWC category *first person pronouns* and (4) less words in the LIWC category *past tense*. In addition, we analyzed associations of these categories with more broadly defined reported momentary affective states (i.e., positive and negative affective state, valence, energetic arousal, calmness). We hypothesized that the associations of LIWC categories with reported negative affective state are in the same direction as those for reported depression severity and in the opposite direction for the remaining reported affective states.

## 2 MATERIALS AND METHODS

Detailed information on the sample, the study procedure and assessment tools can be found in Reference [8] and Appendix S1. Data were collected from 30 inpatients at the Central Institute of Mental Health in Mannheim, Germany, who experienced an acute depressive episode (ICD‐10) at admittance to the hospital. Patients were informed about the study procedures, gave informed consent before being included in the study, and could withdraw at any time. Patients underwent SDT as part of their treatment, which involved staying awake for 36 h. AA collection covered the period before, during, and after SDT (up to 26 days in total). In the first week of the study, 3 e‐diary prompts per day (morning, afternoon, and evening) were send via smartphones, which was reduced to morning and evening prompts after the first week to minimize patient burden. E‐diary prompts included a request to respond to items about the current affective state on (a) the short version of the Center for Epidemiologic Studies Depression Scale (Allgemeine Depressionsskala in German (ADS‐K)^26^), (b) 15 positive and negative affective state items from an item pool,^27^ and (c) a short version of the Multidimensional Mood Questionnaire (MDMQ^28^), and to record a selfie video to report current feelings (“Please describe in 10–20 s how you currently feel.”).

From originally 899 recorded selfie videos we had to exclude 155 files, mostly for technical reasons (see Appendix S1). We obtained transcripts of the audio tracks using an automatic speech recognition system^29^ and corrected them manually. The patients' transcripts were analyzed using the language processing tool LIWC,^11^ a well‐established, transparent and reliable computer text analysis program that automatically classifies words into categories stored in a predefined dictionary.^30^ The output is a percentage of words allocated to the linguistic categories in relation to the total number of words in a text sample. The German LIWC 2015 version used in this study contains 18,711 words and word stems in more than 80 word categories.^30^ We focus on four LIWC categories: positive emotion words (*posemo*), negative emotion words (*negemo*), first person singular pronouns (*I*), and past tense (*focuspast*).

To analyze the within‐subject association of LIWC categories and reported momentary affective states, we applied multilevel modeling^31^ with person centered time‐variant level‐1 predictors (LIWC categories) and added the predictors time and time^2^ in hours (centered at 2 p.m.) as covariates. We calculated separate models for each LIWC category: *positive emotion words*, *negative emotion words*, *first person singular*, *past tense*, and each affective state as outcome (*reported depression severity* (ADS‐K), *positive affective state*, *negative affective state*, *valence*, *energetic arousal*, *calmness*), resulting in 24 models. As the number of speech samples was limited, we included all reported momentary affect ratings and speech recordings available irrespective of the time of assessment. We set the initial α level to 5%, applied Bonferroni corrections construct‐wise (*α*adj = 0.008), and performed all analyses in R (version 4.3.1 [16 June, 2023]). See^8^ for details of statistical parameters and used R packages.

## 3 RESULTS

In the final analysis, we included 744 pairs of sampled speech and affective states, an average of 34 ± 20.16 pairs per person (range = 5–87). Descriptive statistics on word categories and reported affective states, ICCs, and reliability indices (McDonald Omega and Spearman‐Brown coefficients) are presented in Table 1. The upper part depicts the four LIWC categories analyzed, starting with the total word count of all speech samples with 28,128 words. Hereafter follow the four LIWC categories with the highest percentage of words found for *first person singular pronouns* (10.81%), followed by *positive emotion words* (6.22%), *negative emotion words* (4.64%), and *past focus* (3.72%). The amount of between‐person variance represented by the ICCs (0.14–0.32) indicates that most of the variance is within‐subject, strongly arguing for the chosen assessment and analysis approach. The lower part of Table 1 refers to the reported affective momentary states. Mean reported depression severity (ADS‐K) is presented for all assessment points (1.2 ± 0.3) and for the baseline assessment only (1.5 ± 0.3), both to be classified as medium high on a scale from 0 to 3. Negative (2.4 ± 0.5) and positive (2.1 ± 0.4) affect items were medium high on average (scale 1–5), as well as MDMQ scores (valence: 44.7 ± 10.8; energetic arousal: 41.6 ± 10.6; calmness: 43.4 ± 11.45; scale 1–100). The ICCs (0.26–0.60) encourage us to have sufficient within‐person variance to run multilevel regression analysis. The reliability indices were good to excellent as evaluated according to McDonald Omega and Spearman‐Brown coefficients.

**TABLE 1:** Descriptive statistics of Linguistic Inquiry and Word Count (LIWC) categories and momentary affective states.

Category/variable	Output label/scale	Examples	Total number of words	Mean (%) (SD; min^a^, max^a^)	ICC	Reliability (within)	Reliability (between)
LIWC							
Total word count	WC		28,128				
Past focus words	Focuspast	Yesterday, said, was	1142	3.72 (3.87; 0, 7.54)	0.14		
Positive emotion words	Posemo	Happy, nice, good	1609	6.22 (4.21; 0.48, 9.24)	0.17		
Negative emotion words	Negemo	Sad, fear, nervous	1139	4.64 (4.87; 1.63, 14.98)	0.32		
First person singular pronouns	I	I, me, mine	3009	10.81 (4.79; 7.03, 14.81)	0.21		
Affective states							
ADS‐K	0–3			1.2 (0.6; 0.7, 2.0)	0.48	0.87	0.90
ADS‐K (baseline)	0–3			1.5 (0.6; 0.4, 2.7)			
Negative affective state	1–5			2.4 (1.0; 1.4, 3.9)	0.60	0.87	0.96
Positive affective state	1–5			2.1 (0.8; 1.3, 3.2)	0.47	0.87	0.95
Valence	1–100			44.7 (21.6; 9.4, 67.5)	0.26	0.82	0.92
Energetic arousal	1–100			41.6 (21.2; 16.4, 62.7)	0.29	0.61	0.84
Calmness	1–100			43.4 (22.9; 9.4, 67.5)	0.41	0.74	0.91

^a^ The mean of all patients' minimum and maximum scores.

**FIGURE 1:**
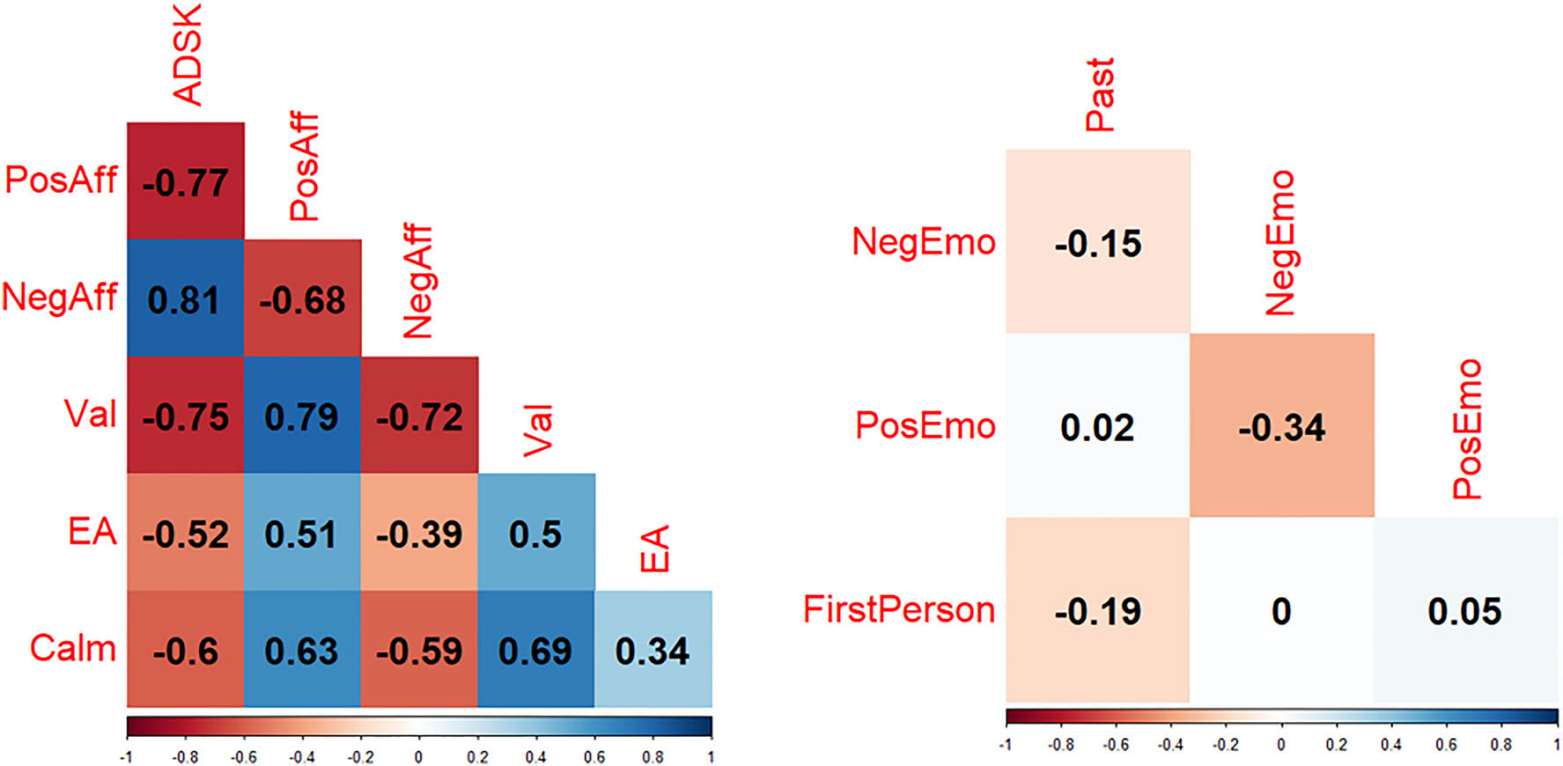
Pearson correlations with person‐mean‐centered variables between affective states and between LIWC categories (n between 729 and 744). Calm, calmness; EA, energetic arousal; FirstPerson, first person singular pronoun; NegAff, negative affective state; NegEmo, negative emotion words; Past, past tense; PosAff, positive affective state; PosEmo, positive emotion words; Val, valence.

In Figure 1, we illustrate Pearson correlation coefficients with person‐mean‐centered scores. The left graph displays correlations of the six affective state variables, the right graph displays correlations of the four LIWC categories. The color scheme encodes the direction and the strength of the correlation; blue indicates a positive correlation with darker blue indicating stronger correlations; red reflects the same pattern but for negative correlations. On the y‐axis and the diagonal, the names of the respective variable pairs are depicted.

Regarding correlational analyses, two main patterns emerged (Figure 1): The e‐Diary items on affective states showed a high coherence while the LIWC categories were weakly correlated. In detail, we found strong positive correlations (*r* > 0.5) between *reported depression severity* and *negative affective state*; strong negative correlations between *reported depression severity* and *positive affective state*, and all MDMQ subscales. The same was found for *negative affective state* except for a moderately strong negative correlation with *energetic arousal*. Correlations between *positive affective state* and other momentary states were opposite to the pattern found for the *reported depression severity* (all strong). *Valence* correlated strongly positively with *energetic arousal* and *calmness*. *Calmness* and *energetic arousal* correlated moderately in a positive way.

Regarding LIWC features, *positive* and *negative emotion words* showed the highest (negative) correlation, which can be classified as moderate. *Past focus words* correlated weakly with *negative emotion words* and *first person singular pronouns*. The other pairings were not meaningfully linked to each other.

Table 2a represents the fixed effects of the separate multilevel models with *positive* and *negative emotion words*, *first person singular*, and *past focus* as statistical predictors. *Momentary reported depression*, *positive* and *negative affective state* are defined as outcomes. Table 2b has the same structure, but reports outcomes related to MDMQ dimensions *valence*, *energetic arousal* and *calmness*. All models also included the centered time and time^2^ variables as statistical predictors. As findings are comparable, we report the simpler models here and expanded models in the Appendix S1.

**TABLE 2a:** Multilevel linear regression analysis to predict depression and positive and negative affective states: fixed effects of Linguistic Inquiry and Word Count (LIWC) categories.

Statistical predictors: LIWC categories	E‐diary ratings											
	Outcome: ADS‐K				Outcome: positive affective state				Outcome: negative affective state			
	*β*	Stand. *β*	SE	*p* value	*β*	Stand. *β*	SE	*p* value	*β*	Stand. *β*	SE	*p* value
Positive emotion words	−0.02	−0.14	<0.01	**<0.001**	0.03	0.16	<0.01	**<0.001**	−0.02	−0.09	<0.01	**<0.001**
Negative emotion words	0.02	0.16	<0.01	**<0.001**	−0.03	−0.18	<0.01	**<0.001**	0.03	0.15	<0.01	**<0.001**
First person singular	<0.01	0.08	<0.01	0.066	<−0.01	−0.04	<0.01	0.214	0.01	0.07	<0.01	**0.007**
Focus past	<−0.01	−0.03	<0.01	0.298	<0.01	0.05	<0.01	0.135	<−0.01	<−0.01	<0.01	0.876

Bold indicates significant *p* values.

**TABLE 2b:** Multilevel linear regression analysis to predict valence, energetic arousal and calmness: fixed effects of Linguistic Inquiry and Word Count (LIWC) categories.

Statistical predictors: LIWC categories	E‐diary ratings											
	Outcome: valence				Outcome: energetic arousal				Outcome: calmness			
	*β*	Stand. *β*	SE	*p* value	*β*	Stand. *β*	SE	*p* value	*β*	Stand. *β*	SE	*p* value
Positive emotion words	1.12	0.22	0.18	**<0.001**	0.99	0.20	0.17	**<0.001**	1.19	0.22	0.17	**<0.001**
Negative emotion words	−1.24	−0.28	0.17	**<0.001**	−1.11	−0.25	0.15	**<0.001**	−0.95	−0.20	0.16	**<0.001**
First person singular	−0.21	0.05	0.17	0.215	−0.06	−0.01	0.15	0.713	−0.25	−0.05	0.16	0.110
Focus past	0.21	0.04	0.20	0.297	0.25	0.05	0.19	0.181	0.42	0.07	0.19	0.028

Bold indicates significant *p* values.

### 3.1 Positive and negative emotion words

The first two lines in Tables 2a and 2b depict the results for the LIWC categories *positive* and *negative emotion words* as statistical predictors. Overall, there is a coherent pattern of significant associations between these two LIWC categories and momentary affective ratings. Specifically, more words in the category *positive emotion words* were significantly associated with less depressive symptomatology (std. *β* = −0.14), more positive affective state (std. *β* = 0.16), less negative affective state (std. *β* = −0.09), more (positive) valence (std. *β* = 0.22), more energetic arousal (std. *β* = 0.20), and more calmness (std. *β* = 0.22). The associations between *negative emotion words* and the momentary affective states were in the opposite direction of those just presented. Specifically, more words in the category *negative emotion words* were significantly associated with more severe reported depressive symptomatology (std. *β* = 0.16), less positive affective state (std. *β* = −0.18), more negative affective state (std. *β* = 0.15), less (positive) valence (std. *β* = −0.28), less energetic arousal (std. *β* = −0.25), and less calmness (std. *β* = −0.20). Effect sizes approximated with standardized beta values are comparably high with regard to the outcomes positive and negative emotion words.

### 3.2 First person singular words

Next, we show results for the LIWC category *first person singular*, which did not reveal such a coherent pattern as *positive*/ *negative emotion words*. *First person singular* was significantly associated with negative affective state (std. *β* = 0.07). There was a trend with respect to reported depressive symptomatology, but this result was not statistically significant (std. *β* = 0.08). All other associations were not significant.

### 3.3 Past focus words

The LIWC category *past focus* was not significantly associated with any of the momentary affective state variables.

## 4 DISCUSSION

This is the first study to investigate momentary affective states and concomitant speech samples collected longitudinally via AA from patients diagnosed with MDD during SDT inducing rapid shifts in symptomatology. We found a coherent pattern for the LIWC categories *positive* and *negative emotion* words in association with concurrently reported affective states. Specifically, we found that using more positive emotion words and fewer negative ones is linked to lower reported depression severity and negative affective states. Additionally, it corresponds to higher levels of positive affective states, (positive) valence, energetic arousal, and calmness. These results suggest that changes in linguistic style extracted from ambulatorily assessed everyday speech samples may be indicative of mood changes.

As hypothesized, we found a positive association between MDD severity and the frequency of negative emotion words and a negative association between MDD severity and positive emotion words. This replicates previous work^14^, ^15^ and is in line with Beck's depression model,^17^ suggesting that patients suffering from MDD have a severely negative attitude. Interestingly, our results generalized over the more broadly defined affective states assessed (positive and negative affective states, valence, calmness, energetic arousal). This connects to discussions on speech features being considered disorder‐specific or transdiagnostic.^13^, ^16^

With respect to our hypothesis on first person singular pronoun use, our results were more limited. An increased use was observed in association with negative affective states and a trend for depressive symptoms, but not with any of the other variables. In a previous review, a small positive correlation was found indicating higher self‐focus during depressive states.^20^ One possible explanation for the lack of a significant relationship between reported depression and first person singular personal pronouns lies in the nature of the task. Unlike studies that have collected language content during therapy sessions,^15^, ^16^ or free spontaneous speech,^19^ patients in this study talked about their current mood in a selfie video, which might have been beneficial as more mood related content has been reported. Furthermore, earlier studies concluded that linguistic indicators of self‐referencing seem to rather reflect the negative affective component of depressive symptoms. Thus, they might be rather broader indicators of negative affectivity than specifically indicating depressive states.^21^

Our fourth hypothesis, which was based on prior studies showing that MDD patients refer more frequently to the past in their narratives compared to healthy participants,^23^, ^24^ was not supported by our data. However, in a more recent study, more *past focus* words were associated with *less* depression severity.^19^ Surprisingly and not in line with any of these previous findings, in our study the amount of past words was not associated with MDD. It is likely that our task instructions resulted in a rather low variance of past word frequencies between time points and different affective states in contrast to earlier language samples that often instructed a life review feature.^23^

Our results suggest that short speech samples collected in everyday life may serve as a behavioral marker of changes in depression severity and may to some extent compensate for the lack of objective biomarkers in future studies. The use of emotion words in particular (positive and negative) may provide clinically meaningful information that could contribute to the detection of impending episodes. Taking the advantage of the widespread availability of smartphones and smartwatches that people wear every day, speech‐based monitoring of MDD is a promising approach. It should be noted that we consider such an everyday speech tool to be a supportive monitoring system, coexisting with clinical sessions which are essential. However, due to its continuous and unobtrusive applicability, speech‐based monitoring could offer the crucial advantage of bridging the time between clinical sessions. Patients who are approaching their personal relapse threshold could be identified earlier, for examp;e, if their linguistic style shows the use of more negative emotion words.

While promising in theory, the development of a speech‐based monitoring system faces many challenges. We have to identify a speech sampling strategy that is (a) most informative, (b) comes with the smallest patient burden, (c) preserves the privacy of patients and bystanders, and (d) impacts smartphone battery life minimally. More specifically, it is unclear whether active sensing (i.e. asking patients to actively record a speech sample or call a system) is necessary or whether passive sensing is sufficient. Especially in the case of passive sensing, the need for privacy‐preserving tools requires technological development, for example, to filter out bystander's speech or to create transcripts in real‐time.

This pilot study had several limitations. First, the sample size was limited and future studies are needed to investigate replicability of results as well as generalizability beyond an inpatient sample. Still, the dataset at hand is unique as it is based on a true within‐subject design with a relatively high number of data points per patient. A meaningful amount of variability in reported depression severity over a short period of time is stimulated by the study design; this is crucial from a theoretical perspective, as meaningful variance is necessary in both parameters to uncover existing associations. Second, SDT might have additional effects, such as fatigue, beyond the antidepressant one. This has to be explored in future work. Third, as we focused our analysis on four LIWC categories, it remains unclear whether other word categories are also sensitive to changing depressive states. However, as a vast variety of possible linguistic features is assess‐ and extractable in theory, feature selection should be made in a considered manner in order to limit alpha error inflation and to increase replicability.^32^ We decided to focus on features resembling psychopathological phenomena in MDD as closely as possible and for which both a theoretical foundation and previous empirical evidence are available. Fourth, LIWC is a word count based automated language analysis and does not consider the context in which the categorized words are used or negations. However, recent findings show comparability between LIWC‐based and hand‐labeled (and thus “corrected”) categories.^22^ LIWC categories can be compared across studies and have thus proven to be reliable and valid.^21^ Fifth, although we used automated transcription, its manual correction was time‐intense. For the future, especially if linguistic analysis is used in the context of AA, it is crucial to have reliable speech‐to‐text tools, as manual correction won't be feasible. Finally, our speech task instruction might have biased word use. Whereas this might have increased number of negative and positive words, it might have limited words related to the past. While previous LIWC studies mostly collected longer speech samples or narratives, we requested brief samples to reduce patient burden. Still identifying significant associations between linguistic style and reported depression severity supports the reliability of the measurement and is promising for AA speech sample collection, where minimizing effort per assessment is crucial for feasibility.

To conclude, our study provides evidence for associations between fluctuations in the use of positive and negative emotion words and momentary affective states. These changes happened within a relative short time, not lagging behind and as such are a real marker. We want to particularly emphasize that the sample consisted of clinically diagnosed patients with an acute depressive episode. The intervention study design involving SDT ensured a maximum of within subject dynamics of affective states within just a few days. The use of these words as a marker is promising for the development of future technology predicting upcoming episodes on an individual level. And this research adds important observations with respect to the aim of developing an automated depression monitoring system.

## AUTHOR CONTRIBUTIONS

Marcella Rietschel, Jerome C. Foo, Josef Frank, Stephanie H. Witt, Lea Sirignano, Maria Gilles, Fabian Streit, Ulrich W. Ebner‐Priemer planned the investigation and developed the sampling scheme. Maria Gilles, Lea Sirignano were responsible for data collection. Lisa‐Marie Hartnagel preprocessed the data, carried out statistical analysis, interpreted results and drafted the manuscript. Matthias F. Limberger, Ulrich W. Ebner‐Priemer and Lea Sirignano contributed to analysis, interpretation and the drafting of the manuscript. Andrea B. Horn contributed to Linguistic Inquiry and Word Count analyses. All authors revised and edited the manuscript critically and had final approval of the version to be published.

## FUNDING INFORMATION

This work was funded by the Deutsche Forschungsgemeinschaft (DFG, German Research Foundation)—GRK2739/1—Project No. 447089431—Research Training Group: KD^2^School—Designing Adaptive Systems for Economic Decisions; ERA‐NET NEURON “EMBED‐impact of Early life MetaBolic and psychosocial strEss on susceptibility to mental Disorders; from converging epigenetic signatures to novel targets for therapeutic intervention” [01EW1904]; supported in part by the DFG Project No. 402170461 [TRR 265 “Losing and Regaining Control over Drug intake”] and the Federal Ministry of Research and Education (BMBF) [RELATER 01EF1803B: Improving communication in psychiatric care of refugees using mobile technology].

## CONFLICT OF INTEREST STATEMENT

Ulrich W. Ebner‐Priemer reports consultancy for Boehringer‐Ingelheim and speaker honorarium from Angelini Pharma, which both had no influence on the content of this article. All other authors declare no conflict of interest.

## ETHICS STATEMENT

The Ethics Committee II of the Medical Faculty Mannheim, University of Heidelberg, approved the study protocol.

## Supporting information


**Appendix S1:** Supporting Information.

## Data Availability

The data that support the findings of this study are available on request from the corresponding author. The data are not publicly available due to privacy or ethical restrictions.
