# Supplementary material for: Linguistic style as a digital marker for depression severity: An ambulatory assessment pilot study in patients with depressive disorder undergoing sleep deprivation therapy
Source: Acta Psychiatr Scand. 2024 Jul 10;151(3):348–57. doi: 10.1111/acps.13726 (PMC11787911; doi:10.1111/acps.13726)
Supplement: Supplementary file 1 — Appendix S1: Supporting Information. [file ACPS-151-348-s001.pdf]

Supporting Information

<u>Reasons for exclusion of selfie videos.</u>

- the full set of videos from four patients
    - one patient did not say anything during the videos (23 files)
    - in the videos of two patients no sound was recorded due to technical issues (30 files)
    - one patient provided only 2 videos (2)
- videos with technical damages (2)
- test runs (14)
- accidental recordings without content (30)
- files in which the microphone was masked (16)
- assessments in which either the speech sample or the reported affective states were missing (19)
- files of consecutive assessments less than 15 minutes apart from each other (19); here only the first assessment was kept unless its audio quality was insufficient or only the second assessment included assessments of affective states; in such cases the second assessment was kept

ADS-K items with English translations in italics.

1) Während der letzten Minuten haben mich Dinge beunruhigt, die mir sonst nichts ausmachen.
During the last few minutes, things that normally don't bother me worried me.

2) Während der letzten Minuten konnte ich meine trübsinnige Laune nicht loswerden, obwohl mich meine Freunde / Familie / Mitpatienten versuchten aufzumuntern.
During the last few minutes, I couldn't get rid of my gloomy mood, although my friends / family / fellow patients tried to cheer me up.

3) Während der letzten Minuten hatte ich Mühe mich zu konzentrieren.
During the last few minutes I had trouble concentrating.

4) Während der letzten Minuten war ich deprimiert / niedergeschlagen.
During the last few minutes I was depressed / down.

5) Während der letzten Minuten war alles anstrengend für mich.
During the last minutes everything was exhausting for me.

6) Während der letzten Minuten dachte ich, mein Leben ist ein einziger Fehlschlag.
During the last minutes I thought my life was one big failure.

7) Während der letzten Minuten hatte ich Angst.
During the last minutes I was afraid.

8) Während der letzten Minuten war ich fröhlich gestimmt.
During the last minutes I was in a cheerful mood.

9) Während der letzten Minuten habe ich weniger als sonst geredet.
During the last minutes I talked less than usual.

10) Während der letzten Minuten fühlte ich mich einsam.
During the last minutes I felt lonely.

11) Während der letzten Minuten habe ich das Leben genossen.
During the last minutes I enjoyed life.

12) Während der letzten Minuten war ich traurig.
During the last minutes I felt sad.

13) Während der letzten Minuten hatte ich das Gefühl, dass mich die Leute nicht leiden können.
During the last minutes I felt that people didn't like me.

14) Während der letzten Minuten konnte ich mich zu nichts aufraffen.
During the last minutes I couldn't get myself up to do anything.

MDMQ items with English translations in italics.

Im Moment fühle ich mich …
At the moment I feel ...

unzufrieden – zufrieden
discontent- content

unwohl – wohl
unwell - well

müde – wach
tired - awake

Im Moment fühle ich mich …
At the moment I feel ...

energielos – energiegeladen
without energy - full of energy

unruhig – ruhig
agitated - calm

angespannt – entspannt
tense - relaxed

Positive and negative affect items with English translations in italics.

Im Moment fühle ich mich
fröhlich / zufrieden / tatkräftig / enthusiastisch / entspannt / glücklich; einsam / traurig / unsicher / ängstlich / niedergeschlagen / schuldig / deprimiert / misstrauisch / gereizt

At the moment I feel
cheerful / content / energetic / enthusiastic / relaxed / happy;
lonely, sad, insecure, anxious, depressed, low-spirited, guilty, distrustful, irritable

Multilevel linear regression analysis to predict momentary depression severity and affective states: fixed effects of the LIWC categories positive emotion words, negative emotion words, first person singular pronouns, past tense and time and time².

Outcome	Fixed				
Statistical Predictor	Beta Coefficient	Standardized Beta Coefficient	Standard Error	T	*P*-Value
**ADS-K**					
Intercept	1.27		0.10	12.93	<.001
Time	<0.01		<0.01	0.21	.83
Time-squared	<-0.01		<0.01	-0.20	.84
Positive Emotion Words	-0.02	-0.14	<0.01	-4.67	<.001
**Positive affective state**					
Intercept	2.10		0.13	16.53	<.001
Time	<-0.01		<0.01	-0.79	.43
Time-squared	<-0.01		<0.01	-0.22	.83
Positive Emotion Words	0.03	0.16	<0.01	5.69	<.001
**Negative affective state**					
Intercept	2.46		0.17	14.73	<.001
Time	<0.01		<0.01	1.41	.16
Time-squared	<-0.01		<0.01	-1.73	.08
Positive Emotion Words	-0.02	-0.09	<0.01	-4.00	<.001
**Valence**					
Intercept	43.49		2.81	15.50	<.001
Time	0.17		0.13	1.32	.19
Time-squared	0.04		0.03	1.37	.17
Positive Emotion Words	1.12	0.22	0.18	6.09	<.001
**Energetic arousal**					
Intercept	42.58		2.82	15.08	<.001
Time	-0.42	-0.11	0.12	-3.47	<.001
Time-squared	-0.12	-0.16	0.02	-4.78	<.001
Positive Emotion Words	0.99	0.20	0.17	5.91	<.001

**Calmness**					
Intercept	40.45		3.48	11.62	<.001
Time	<0.01		0.12	0.03	.99
Time-squared	0.07	0.09	0.02	2.65	.008
Positive Emotion Words	1.19	0.22	0.17	7.02	<.001
**ADS-K**					
Intercept	1.27		0.10	12.95	<.001
Time	<0.01		<0.01	0.52	.60
Time-squared	<-0.01		<0.01	-0.15	.88
Negative Emotion Words	0.02	0.16	<0.01	6.13	<.001
**Positive affective state**					
Intercept	2.11		0.13	16.54	<.001
Time	<-0.01		<0.01	-1.14	.26
Time-squared	<-0.01		<0.01	-0.32	.75
Negative Emotion Words	-0.03	-0.18	<0.01	-6.61	<.001
**Negative affective state**					
Intercept	2.46		0.17	14.73	<.001
Time	<0.01		<0.01	1.72	.085
Time-squared	<-0.01		<0.01	-1.70	.090
Negative Emotion Words	0.03	0.15	<0.01	5.69	<.001
**Valence**					
Intercept	43.57		2.81	15.52	<.001
Time	0.13		0.13	0.98	.33
Time-squared	0.03		0.03	1.26	.21
Negative Emotion Words	-1.24	-0.28	0.17	-7.31	<.001
**Energetic arousal**					
Intercept	42.64		2.83	15.09	<.001
Time	-0.46	-0.12	0.12	-3.85	<.001
Time-squared	-0.12	-0.16	0.02	-4.96	<.001
Negative Emotion Words	-1.11	-0.25	0.15	-7.15	<.001

**Calmness**					
Intercept	40.55		3.48	11.64	<.001
Time	-0.03		0.12	-0.25	.801
Time-squared	0.06	0.07	0.02	2.48	.014
Negative Emotion Words	-0.95	-0.20	0.16	-5.93	<.001
**ADS-K**					
Intercept	1.27		0.10	12.85	<.001
Time	<0.01		<0.01	0.17	.87
Time-squared	<0.01		<0.01	0.05	.96
First person pronoun	<0.01	0.08	<0.01	1.84	.07
**Positive affective state**					
Intercept	2.11		0.13	16.56	<.001
Time	<-0.01		<0.01	-0.73	.47
Time-squared	<-0.01		<0.01	-0.48	.63
First person pronoun	<-0.01	-0.04	<0.01	-1.24	.21
**Negative affective state**					
Intercept	2.45		0.17	14.67	<.001
Time	<0.01		<0.01	1.36	.17
Time-squared	<0.01		<0.01	-1.43	.15
First person pronoun	0.01	0.07	<0.01	2.73	.007
**Valence**					
Intercept	43.66		2.80	15.59	<.001
Time	0.18		0.14	1.32	.19
Time-squared	0.03		0.03	1.13	.26
First person pronoun	-0.21	-0.05	0.17	-1.24	.22
**Energetic arousal**					
Intercept	42.71		2.83	15.10	<.001
Time	-0.42	-0.11	0.12	-3.36	<.001
Time-squared	-0.12	-0.16	0.02	-4.83	<.001
First person pronoun	-0.06	-0.01	0.15	-0.37	.71

**Calmness**					
Intercept	40.65		3.49	11.67	<.001
Time	<0.01		0.13	0.06	.95
Time-squared	0.06	0.07	0.03	2.32	.021
First person pronoun	-0.25	-0.05	0.16	-1.60	.11
**ADS-K**					
Intercept	1.27		0.10	12.87	<.001
Time	<0.01		<0.01	0.20	.84
Time-squared	<-0.01		<0.01	-0.03	.98
Past tense	<-0.01	-0.03	<0.01	-1.04	.30
**Positive affective state**					
Intercept	2.11		0.13	16.56	<.001
Time	<-0.01		<0.01	-0.77	.44
Time-squared	<-0.01		<0.01	-0.45	.65
Past tense	<0.01	0.05	<0.01	1.50	.13
**Negative affective state**					
Intercept	2.45		0.16	14.70	<.001
Time	<0.01		<0.01	1.37	.17
Time-squared	<-0.01		<0.01	-1.56	.12
Past tense	<-0.01	<-0.01	<0.01	-0.16	.88
**Valence**					
Intercept	43.62		2.80	15.58	<.001
Time	0.17		0.14	1.29	.20
Time-squared	0.03		0.03	1.18	.24
Past tense	0.21	0.04	0.20	1.04	.30
**Energetic arousal**					
Intercept	42.70		2.83	15.10	<.001
Time	-0.42	-0.11	0.12	-3.40	<.001
Time-squared	-0.12	-0.16	0.02	-4.83	<.001
Past tense	0.25	0.05	0.19	1.34	.18

**Calmness**					
Intercept	40.60		3.48	11.65	<.001
Time	<-0.01		0.13	<-0.01	.99
Time-squared	0.06	0.06	0.03	2.38	.017
Past tense	0.42	0.07	0.19	2.20	.028
